# Comparison of Hybrid capture 2 testing at different thresholds with cytology as primary cervical screening test

**DOI:** 10.1038/sj.bjc.6605869

**Published:** 2010-08-31

**Authors:** D C Rijkaart, V M H Coupe, F J van Kemenade, D A M Heideman, A T Hesselink, W Verweij, L Rozendaal, R H Verheijen, P J Snijders, J Berkhof, C J L M Meijer

**Affiliations:** 1 Department of Pathology, VU University Medical Center, P.O. Box 7057, Amsterdam 1007 MB, The Netherlands; 2 Department of Epidemiology and Biostatistics, VU University Medical Center, Amsterdam, The Netherlands; 3 SALTRO, Primary Health Care Laboratory, Utrecht, The Netherlands; 4 Division of Woman and Baby, Department of Gynaecological Oncology, University Medical Center, Utrecht, The Netherlands

**Keywords:** cervical cancer, cervical cancer screening, HPV DNA testing, cytology, Hybrid Capture 2, triage

## Abstract

**Background:**

We evaluated the performance of primary high-risk human papillomavirus (hrHPV) testing by hybrid capture 2 (HC2) with different thresholds for positivity, in comparison with conventional cytology.

**Methods:**

We used data of 25 871 women (aged 30–60 years) from the intervention group of the VUSA-Screen study (VU University Medical Center and Saltro laboratory population-based cervical screening study), who were screened by cytology and hrHPV. Primary outcome measure was the number of cervical intraepithelial neoplasia grade 3 or higher (CIN3+), detected within 3 years. We compared baseline cytology testing with three possible hrHPV screening strategies at different relative light unit/cutoff (RLU/CO) thresholds.

**Results:**

Compared with baseline cytology testing, hrHPV DNA testing as a sole primary screening instrument did not yield a superior sensitivity, as well as lower colposcopy referral rate and lower false positivity rate at any RLU/CO threshold. The hrHPV screening at 1 RLU/CO threshold with cytology triage at baseline and at 12 months revealed the highest sensitivity for CIN3+ (relative sensitivity of 1.32), although still displaying a lower colposcopy referral rate than cytology testing (relative colposcopy rate of 0.94). Higher thresholds (>1RLU/CO) yielded lower colposcopy rates, but resulted in substantial loss in sensitivity.

**Conclusions:**

The hrHPV testing at the commonly used threshold of 1 RLU/CO with cytology triage at baseline and at 12 months showed a much higher sensitivity with a lower colposcopy referral rate compared with cytology testing.

New promising methods of cervical cancer prevention have been introduced since the recognition that infection with high-risk human papillomavirus (hrHPV) is the necessary cause of cervical cancer (Walboomers *et al*, 1999; Bosch *et al*, 2002; Munoz *et al*, 2003). The recently introduced prophylactic HPV vaccine may have a major impact on preventing this global disease. The prophylactic vaccines have shown to be highly effective in preventing premalignant lesions (Harper *et al*, 2006; FUTURE II Study Group, 2007; Paavonen *et al*, 2009; Romanowski *et al*, 2009). However, it is generally agreed upon that cervical cancer screening will need to continue even for vaccinated women (Franco *et al*, 2006; Goldhaber-Fiebert *et al*, 2008; Coupe *et al*, 2009).

Although cytological screening has reduced the incidence and mortality of cervical cancer (Bray *et al*, 2005), it has a limited sensitivity. The much more sensitive hrHPV test has been suggested as an alternative primary screening instrument (Cuzick *et al*, 2006; Bulkmans *et al*, 2007; Mayrand *et al*, 2007; Naucler *et al*, 2007; Ronco *et al*, 2008, 2010), given that a clinically validated hrHPV assay is used (Meijer *et al*, 2009). At present, the FDA approved hybrid capture 2 (HC2) assay is most commonly used. However, hrHPV testing using such a test has also shown a 4–6% lower specificity than conventional cytology (Arbyn *et al*, 2006; Cuzick *et al*, 2006), because many detected infections are transient and regress without developing high-grade lesions.

In population-based screening, specificity is of utmost importance, as it basically determines the costs of the programme and the amount of unwanted adverse effects (anxiety, repetitive and confirmatory tests, as well as unnecessary colposcopy referrals and treatments) in the generally healthy population. Cytological triage of hrHPV DNA-positive women has been found to improve the specificity of the screening test (Cuzick *et al*, 2006; Ronco *et al*, 2006b; Naucler *et al*, 2009). Another, easier and potentially cost saving option is to adapt the threshold, that is, increase the relative light unit/cutoff (RLU/CO) threshold, of the HC2 test. This would particularly be useful if it obviates the need for repeat testing. Several studies have examined baseline hrHPV testing strategies with triage of hrHPV-positive women at higher thresholds than the one conventionally used (i.e., RLU/CO of ⩾1) (Clavel *et al*, 2001; Hesselink *et al*, 2006; Kotaniemi-Talonen *et al*, 2008; Ronco *et al*, 2008; Sargent *et al*, 2010). This did not result in a strategy that ensured higher sensitivity as well as higher specificity in terms of lower colposcopy referral rates compared with cytological screening.

The aim of this study was to investigate the effect of hrHPV HC2 testing with higher thresholds on the sensitivity and specificity in terms of colposcopy referral rate and false positivity rate, considering a number of different hrHPV screening strategies. We searched for strategies that improved the specificity of hrHPV screening by increasing the RLU/CO threshold, while maintaining a higher sensitivity than baseline cytological testing. We used data from the intervention group of the VUSA-Screen study (VU University Medical Center and Saltro laboratory population-based cervical screening study), a study performed within the routine cervical programme of the Netherlands. Women participating in this cohort received combined hrHPV testing and cytology. The primary end points were cervical intraepithelial neoplasia grade 3 or higher (CIN3+), detected within 3 years.

## Materials and methods

### Study design VUSA-screen

The VUSA-Screen is a population-based study designed to evaluate the effectiveness of combined cervical cytology screening with hrHPV testing by HC2 hybridisation assay (Qiagen, Gaithersburg, MD, USA). The study was carried out in the Utrecht province of the Netherlands in the setting of the regular screening programme that invites women, aged between 30 and 60 years of age, to be screened every 5 years. The design of the study has also been described elsewhere (Rijkaart *et al*, 2009). Between October 2003 and August 2005, women invited for the regular cervical screening programme were asked to participate in the VUSA-Screen study. Women were excluded from the analysis if they had a history of cervical intraepithelial neoplasia grade 2 or higher (CIN2+) or abnormal cytology in the preceding 2 years. Women who agreed to receive cytology and hrHPV testing gave written informed consent.

Conventional cytological smears were taken with a cytobrush (Rovers, Oss, The Netherlands). After preparation of a conventional smear on a glass slide, the brush was placed in a vial containing 1 ml UCM (Universal Collection Medium, Digine Corp., Gaithersburg, MD, USA) for hrHPV testing. Cervical cytology results were reported, blinded to the hrHPV testing results, according to the CISOE-A classification, which is routinely used in The Netherlands and can be converted into the 2001 Bethesda system (Bulk *et al*, 2004). Cytological results were grouped as normal, BMD (borderline or mild dyskaryosis) and >BMD (moderate dyskaryosis or worse). In the 2001 Bethesda system, BMD corresponds to atypical squamous cells of undetermined significance; atypical squamous cells cannot rule out high-grade squamous intraepithelial lesions; or low-grade squamous intraepithelial lesions and >BMD corresponds to high-grade squamous intraepithelial lesions.

**Figure 1:**
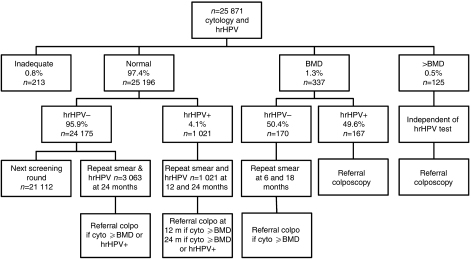
Flowchart of the study design. BMD, borderline or mild dyskaryosis; colpo, colposcopy; cyto, cytology; hrHPV, high-risk human papillomavirus; m, months.

Women with BMD or worse were informed about the hrHPV test result. The hrHPV-positive women with BMD and all women with >BMD were directly referred for colposcopy (Figure 1). Women with BMD at baseline and a negative hrHPV test were offered cytology testing at 6 and 18 months and referred if cytology was abnormal (threshold BMD) at one of these occasions.

In the women with normal cytology at baseline, a sub-study was carried out. In this sub-study, all (*n*=1021) hrHPV-positive women as well as a subset of hrHPV-negative cytologically normal women (*n*=3063) were included. To select the hrHPV-negative women, each hrHPV-positive woman was matched to three randomly chosen hrHPV-negative women of the same age. Women with normal cytology were not informed about the hrHPV test result. The hrHPV-positive women with normal cytology were offered cytology and a blinded hrHPV test at 12 months, and combined hrHPV testing and cytology at 24 months. A woman was referred at 12 months if cytology was abnormal and at 24 months if the hrHPV test was positive and/or cytology was abnormal. The hrHPV-negative, cytologically normal women in the sub-study were invited for combined testing at 24 months, and referred if cytology was abnormal and/or the hrHPV test was positive. If a woman with normal cytology and a negative hrHPV test was not invited for repeat testing after 24 months, cytological and/or histological follow-up results was not included.

The VUSA-Screen study was approved by the Ministry of Public Health (2002/02-WBO; ISBN-10: 90-5549-452-6) and registered in the trial register (NTR215, ISRCTN64621295).

### Colposcopy

Of the women who were referred to a gynaecologist for colposcopy, colposcopy-directed biopsies were taken from suspicious areas of the cervix, according to standard procedures in the Netherlands (Hopman *et al*, 2000). Biopsy results were reported as normal, cervical intraepithelial neoplasia grade 1, 2, 3, or as invasive cancer, according to the international criteria (Anderson, 1995; Wright, 2009). Cytology and histology results were retrieved from the nationwide network and registry of histopathology and cytopathology (PALGA; Bunnik, The Netherlands).

### hrHPV testing

The hrHPV testing was performed by the HC2 high-risk HPV DNA test in an automated format on a rapid capture system according to the manufacturer's instructions (Qiagen). This test uses a cocktail probe to detect 13 high-risk HPV types: 16, 18, 31, 33, 35, 39, 45, 51, 52, 56, 58, 59 and 68. Positive controls containing 1 pg ml^−1^ of cloned HPV-16 DNA and negative controls (provided by the manufacturer) were included in each assay (Qiagen). The results of the HC2 assay were expressed as RLU/CO ratio, representing the ratio between the emission from a sample to the average emission of three positive controls. Initially, the threshold of 1 RLU/CO, as proposed by the manufacturer, was used to classify a specimen as positive or negative.

### Statistical analysis

The primary outcome measure of the study was histologically confirmed CIN3+, detected cumulatively within 3 years after baseline. A secondary outcome was cumulatively detected CIN2+. In the calculations of the number of CIN3+ and CIN2+ lesions, cases of cervical adenocarcinoma and cervical adenocarcinoma *in situ* were also included.

The absolute specificity of hrHPV testing with RLU/CO thresholds between 1 and 100 and the absolute specificity of cytology were computed as follows. Specificities were adjusted for non-attendance at repeat testing by applying Bayes's rule, which means that the specificities were computed from the positive and negative predictive values for CIN3+ (and CIN2+), as well as the baseline prevalences of HC2 and cytology test outcome strata (Begg and Greenes, 1983; Kulasingam *et al*, 2002). For this purpose, the baseline test outcomes were grouped into seven strata: (1) >BMD and hrHPV+, (2) >BDM and hrHPV−, (3) BMD and hrHPV+, (4) BMD and hrHPV−, (5) normal and hrHPV+, (6) normal and hrHPV−, and age ⩽35 years, and (7) normal and hrHPV−, and age >35 years. We defined two separate age strata for hrHPV-negative women with normal cytology at baseline because hrHPV-negative normal women were age-matched to hrHPV-positive normal women, and women ⩽35 years were therefore overrepresented in follow-up. The positive and negative predictive values were computed only on the basis of women with at least one repeat test. For hrHPV-positive, cytologically normal women, the 12-month screening tests were used as repeat tests. The 24-month screening tests were used if the 12-month tests were missing. For BMD hrHPV-negative women, the 6-month results were used as repeat test results and the 18-month results were used if the 6-month results were missing. The specificities presented were therefore adjusted for women without repeat testing, but were not adjusted for differences in intensity of follow-up testing among women with at least one repeat test.

Furthermore, we compared baseline cytology (threshold BMD) with three possible hrHPV screening strategies at RLU/CO thresholds between 1 and 100. The following hrHPV screening strategies were used: (1) baseline hrHPV testing only; (2) cytology triage of hrHPV-positive women at baseline and one repeat cytological test for cytologically normal women; and (3) cytology triage of hrHPV-positive women at baseline and one repeat combined cytology and hrHPV HC2 test (with RLU/CO values as used at baseline) for cytologically normal women. For each comparison, we computed the relative sensitivity for CIN3+ (and CIN2+), relative false positivity rate and relative colposcopy referral rate. Analogous to the calculation of the specificity, the relative rates were calculated by combining positive and negative predictive values (here for CIN3+, CIN2+ and colposcopy referral) and baseline test outcomes. Because double-negative women cancel out when calculating relative rates (Pepe and Alonzo, 2001), we only needed to define five baseline strata: (1) >BMD and hrHPV+, (2) >BDM and hrHPV−, (3) BMD and hrHPV+, (4) BMD and hrHPV− and (5) normal and hrHPV+.

The 95% confidence intervals (CIs) were calculated for absolute specificity using the Wilson Score method (Brown *et al*, 2001), in which the sample size was set equal to the number of cases observed in the cohort study.

Analyses were done with SPSS version 15.0 (LEAD Technologies Inc, Haddonfield, NJ, USA), and Excel (Microsoft Corporation, Redmond, WA, USA).

## Results

### Study subjects

Of the 25 871 women from the intervention group of VUSA-Screen study, 25 658 (99.2%) had an adequate baseline Pap smear. Among women with adequate Pap smears, 25 196 had normal cytology of whom 1021 (4.1%) tested hrHPV-positive, 337 women had a BMD result of whom 167 (49.6%) tested hrHPV-positive and 125 women had a >BMD result of whom 115 (92.0%) tested hrHPV-positive. The median age of participating women was 44.0 years (range, 29–61 years). The hrHPV results of positive RLU/CO (i.e., RLU/CO ⩾1) showed a mean of 224.5 (range, 1.0–2565.7).

The number of test positives and negatives, CIN3+ and CIN2+ detected, stratified for cytology and HC2 thresholds are presented in Table 1.

Table 2 presents the specificity for detected CIN3+ and CIN2+ lesions for baseline cytology testing (threshold BMD) and for a strategy of primary HC2 testing without follow-up testing at different RLU/CO thresholds. Compared with baseline cytology testing, hrHPV testing at the standard threshold of 1 RLU/CO had a lower specificity for CIN3+ (95.5 *vs* 98.7%) and CIN2+ (95.9 *vs* 98.9%). The specificity for CIN3+ and CIN2+ increased with increasing RLU/CO thresholds. Only at a RLU/CO threshold of 100, hrHPV testing reached the same specificity for CIN2+ (i.e., 98.9%) and CIN3+ (i.e., 98.7%) as cytology.

The relative colposcopy referral rate, relative sensitivity and relative false positivity rate of primary HC2 testing at different RLU/CO thresholds *vs* baseline cytology testing are presented in Table 3. Compared with cytology, hrHPV testing at a threshold of 1 RLU/CO would result in a 2.8-fold higher number of colposcopy referral rates. With increasing HC2 threshold, the relative colposopy referral rates decreased, resulting in a relative rate of 1.9 at 10 RLU/CO and 0.94 at 100 RLU/CO.

At the standard test positivity threshold for HC2 (i.e., 1 RLU/CO), the relative sensitivity of hrHPV was superior to that of cytology, both for CIN3+ (relative sensitivity of 1.36) and CIN2+ (relative sensitivity of 1.50). With increasing HC2 threshold values, the relative sensitivity for CIN3+ decreased, resulting in a relative sensitivity of 1.22 at 10 RLU/CO and 0.80 at 100 RLU/CO. Results were comparable using CIN2+ as outcome measure.

There was no HC2 threshold that resulted in an improved false positivity rate and concomitant colposcopy referral rate, without compromising its sensitivity. In fact, the HC2 threshold (i.e., 100 RLU/CO) at which a lower colposcopy referral rate was reached compared with cytology, also revealed lower sensitivities for CIN3+ and CIN2+ (Table 3). As no strategy of sole hrHPV testing at baseline improved on baseline cytology testing, some form of triage or follow-up testing is required.

Table 4 shows the impact of raising the HC2 threshold in the context of two triage and follow-up strategies for HC2-positive women compared with baseline cytology testing. For the strategy with cytology triage at baseline and at 12 months, HC2 screening at RLU/CO thresholds between 1 and 30 resulted in higher sensitivities for both CIN2+ and CIN3+, compared with baseline cytology testing. This strategy showed lower colposcopy referral rates and false positivity rates at all analysed RLU/CO thresholds (1–100).

For the strategy with cytology triage at baseline and combined cytology and HC2 testing (with the same threshold as used at baseline) at 12 months, only at threshold 30, a lower false positivity and colposcopy rate in combination with higher sensitivities for CIN3+ and CIN2+ than baseline cytology testing was found. However, at RLU/CO 30, the gain in sensitivity compared with cytology was only marginal.

**Figure 2:**
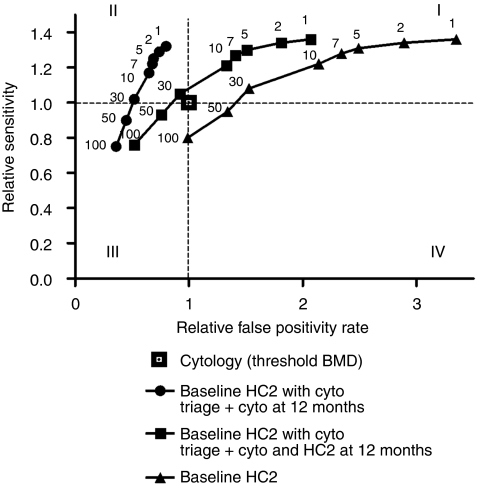
Relative sensitivity *vs* relative false positivity rate for three strategies for hybrid capture 2 (HC2)-positive women at different relative light unit/cutoff (RLU/CO) thresholds compared with baseline cytology (cyto) testing, for detection of cervical intraepithelial neoplasia grade 3 or higher (CIN3+). Relative sensitivity for detection of CIN3+ is plotted on the *y*-axis, against the relative false positivity rate on the *x*-axis. The used HC2 RLU/CO thresholds are indicated at the respective positions above each plot. I: quadrant with relative sensitivity and relative false positivity rate greater than cytology; II: panel with relative sensitivity greater than and relative false positivity rate lower than cytology; III: panel with relative sensitivity lower than and relative false positivity rate lower than cytology; IV: panel with relative sensitivity and relative false positivity rate lower than cytology. BMD, borderline or mild dyskaryosis.

The relative sensitivity *vs* relative false positivity rate of the three investigated screening strategies, compared with baseline cytology testing, is graphically shown in Figure 2. Baseline cytology testing is presented in the origin (relative sensitivity=1, relative specificity=1). Quadrant II represents combinations of sensitivity and false positivity rates that are superior to baseline cytology testing. The strategy with baseline HC2 testing alone was inferior to baseline cytology testing for all RLU/CO thresholds. The strategy with cytology triage of HC2-positive women and cytology testing at 12 months showed the best combination of relative sensitivity and false positivity rates for RLU/CO between 1 and 30. The RLU/CO data points for this strategy form a steep curve. This indicates that at increasing RLU/CO thresholds, the reduction in false positivity rate in this strategy is relatively small, whereas the decrease in sensitivity is substantial. Thus, low RLU/CO values are required to maintain high sensitivity.

## Discussion

In this study, we evaluated three possible cervical screening strategies that are based on hrHPV testing with different HC2 thresholds and compared them with baseline cytology testing (threshold BMD). We aimed to improve the specificity of hrHPV screening by increasing the RLU/CO threshold, while maintaining a higher sensitivity than baseline cytological testing. The results are based on data from the VUSA-Screen study, a population-based cohort study carried out in the Utrecht province of the Netherlands. We found that compared with baseline cytology testing, there was no HC2 RLU/CO threshold for which a screening strategy of hrHPV testing as a sole primary screening instrument resulted in both superior sensitivity as well as similar (or lower) colposcopy rate and equal (or higher) specificity. As baseline hrHPV testing cannot improve baseline cytology testing, even at increased RLU/CO thresholds, we conclude that some form of triage and follow-up is required in hrHPV screening.

Given that follow-up testing is required in hrHPV-positive women, we searched for a strategy that did not increase colposcopy referral rate compared with cytological testing. A screening strategy that was clearly superior to baseline cytological testing was primary hrHPV screening, with RLU/CO thresholds between 1 and 30 and cytology triage at baseline and repeated cytology testing at 12 months. This strategy was not only more sensitive than baseline cytology testing but also resulted in lower false positivity rates and in fewer colposcopy referrals. Using HC2 RLU/CO thresholds between 1 and 5 results in higher sensitivity (relative sensitivity between 1.32 and 1.25, respectively) and a reduced colposcopy referral rate (between 6 and 16%, respectively) compared with baseline cytology testing. The current threshold of 1 RLU/CO makes optimal use of the superior sensitivity of the hrHPV test for CIN3+/CIN2+, without actually increasing the colposcopy referral rate compared with baseline cytology. The colposcopy referral rate of this strategy is therefore substantially lower than a screening scenario with baseline hrHPV testing only. In addition, a screening strategy with a high sensitivity may allow for extension of the screening interval, which in turn reduces colposcopy referral rates (Berkhof *et al*, 2010).

An important issue in the debate about implementation of hrHPV testing has been the increased adverse effects in terms of unnecessary referrals for colposcopy among women with a positive hrHPV test. The issue of overdiagnosis and overtreatment is of particular importance for women of reproductive age, because it has been shown that the rate of serious obstetrical complications, such as preterm deliveries, low birth weight and premature rupture of the membranes, is increased after excisional treatments for precancerous lesions (Kyrgiou *et al*, 2006). Therefore, there is a need to identify strategies that minimise the need for colposcopy referrals with hrHPV testing, while maintaining its advantage in terms of sensitivity. A number of studies have evaluated the optimisation of cervical screening by studying the different hrHPV HC2 cutoff levels for test positivity (Kuhn *et al*, 2000; Schiffman *et al*, 2000; Clavel *et al*, 2001; Kulmala *et al*, 2004; Ronco *et al*, 2006a, 2006b; Kotaniemi-Talonen *et al*, 2008; Sargent *et al*, 2010). Kotaniemi-Talonen *et al* concluded that when used as a sole screening test, the hrHPV test cutoff level can be increased to 10 RLU/CO. The specificity of hrHPV screening, however, remained lower than that with conventional cytology testing even at the threshold of 10 RLU/CO. This is in line with our findings for the strategy of baseline HC2 testing only. It should be noted that the hrHPV test may compare to be more favourable with cytology in other countries. In the Netherlands and Finland, the specificity of cytology is quite high. This is also the case for other European screening programmes (Cuzick *et al*, 2006), but worldwide the specificity of cytology is highly variable (Nanda *et al*, 2000). Ronco *et al* (2006a, 2006b) proposed only a slight increase of the threshold up to 2.00 RLU/CO when HC2 is used for population-based screening. The same threshold has been proposed by Sargent *et al* (2010). A minimal increase in threshold reflects a preference for a sensitive screening strategy. We found that, in an hrHPV DNA screening strategy with cytology triage and cytology testing at one follow-up visit, an increased sensitivity as well as decreased colposcopy referral rate is possible with RLU/CO thresholds up to 30. Given our observation that hrHPV testing cannot be used as a sole screening instrument and that triage and repeated testing is necessary anyhow, we also prefer a low threshold of 1 to maintain the highest sensitivity.

There are some limitations in our study. In this study, women received cytology and hrHPV testing, and based on both results, women were referred for colposcopy. Therefore, we were not able to compare different RLU/CO thresholds outcomes with current cytology screening programme but only with baseline cytology testing. Furthermore, we adjusted for non-attendance at repeat testing, but the results were not adjusted for differences in intensity of follow-up testing. In addition, actual colposcopies were not reported. Another limitation of our study may be the use of a subjective test, such as cytology, as a triage test for hrHPV-positive women. Awareness of the negative or positive hrHPV test result may affect the criteria for defining cellular abnormalities. However, in this study, the cytotechnicians were not informed about the hrHPV test result. Nevertheless, even in case cytotechnicians were aware of the hrHPV test results, as in a Finnish trial (Leinonen *et al*, 2009), the hrHPV test information only had a small effect on cytology assessment, and therefore on the CIN3+ detection rate and the number of colposcopies. In this context, it may be expected that in the near future molecular biomarkers may be used as objective triage tests of hrHPV-positive women. Suitable candidate novel biomarkers such as HPV mRNA (Molden *et al*, 2005), p16 ink4a (Carozzi *et al*, 2008), methylation markers (Overmeer *et al*, 2008, 2009) or genotyping might further enhance the efficacy of screening with hrHPV DNA (Cuschieri *et al*, 2004). Presently, we are investigating the possible value of such alternative triage tests in hrHPV-positive women and preliminary results show that better results can be obtained than with cytology (Heideman *et al*, 2010).

Our finding that hrHPV testing alone at the predefined assay threshold of 1 RLU/CO had a somewhat lower specificity than cytology for CIN2+ and CIN3+ is consistent with results from other randomised and nonrandomised studies using HC2 testing (Kotaniemi-Talonen *et al*, 2005; Arbyn *et al*, 2006; Cuzick *et al*, 2006, 2008; Ronco *et al*, 2006a, 2006b; Mayrand *et al*, 2007; Kitchener *et al*, 2009; Leinonen *et al*, 2009) or another clinically validated hrHPV test (Bulk *et al*, 2007; Naucler *et al*, 2007). However, compared with these studies, our observed specificity of the hrHPV test was relatively high (i.e., 95.9% (95% CI 95.7–96.2) for CIN2+ and 95.5% (95% CI 95.3–95.8) for CIN3+). At least in part, this difference in specificity estimates may be explained by differences in the design of hrHPV screening studies and study populations. Our study included hrHPV testing combined with cytology. In addition, it was conducted within the setting of an organised cervical screening programme with high invitational coverage and low incidence of cervical cancer.

A strong point of this study is the longitudinal design and the older age range of study participants (30–60 years), which is the age for which hrHPV testing is most widely advocated (Wright Jr *et al*, 2004; Naucler *et al*, 2007; Ronco *et al*, 2009). The study was population-based and part of a routine organised screening activity in a low-risk population, indicating that the results could be implemented in routine practice.

To conclude, no RLU/CO threshold was found for which HC2 testing at baseline resulted in a similar or lower colposcopy referral rate than baseline cytology, while maintaining a higher sensitivity.

Superior combinations of sensitivity and colposcopy rate are possible for HC2 testing with cytology triage at baseline and repeated cytology testing after 1 year. As increasing the RLU/CO threshold only marginally decreases colposcopy referral rate while substantially reducing sensitivity, we suggest maintaining the currently used RLU/CO threshold of 1. This results in more than 30% higher sensitivities for CIN3+ than cytology testing at a 20% lower false positivity rate and 6% lower colposcopy referral rate.

## Figures and Tables

**Table 1:** Number of test positives and negatives, CIN3+ and CIN2+ detected, stratified for cytology and HC2 thresholds

			**Histology**			
			**End point CIN3+**		**End point CIN2+**	
	**Test positive**	**Test negative**	**Detected**	**Missed**	**Detected**	**Missed**
**Test**	***n* (% of total *n*=25 658)**	***n***	***n***	***n***	***n***	***n***
Baseline cytology (threshold BMD)	462 (1.80)	1021	124	27	181	57
						
*HC2 cutoff*						
1	1303 (5.08)	180	146	5	227	11
2	1147 (4.47)	336	144	7	224	14
5	1006 (3.92)	477	141	10	216	22
7	954 (3.72)	529	137	14	212	26
10	877 (3.42)	606	131	20	203	35
30	654 (2.55)	829	118	33	184	54
50	572 (2.23)	911	104	47	162	76
100	433 (1.69)	1050	89	62	133	105

Abbreviations: BMD=borderline or mild dyskaryosis; CIN=cervical intraepithelial neoplasia (grade 2 or 3 or higher); HC2=hybrid capture 2.

**Table 2:** Comparison of specificity between baseline hrHPV test with different RLU/CO thresholds and baseline cytology testing for CIN3+ and CIN2+, adjusted for non-attendance at repeat testing

	**End point CIN3+**		**End point CIN2+**	
**Test**	**Specificity (%)**	**95% CI**	**Specificity (%)**	**95% CI**
Baseline cytology (threshold BMD)	98.7	98.5–98.8	98.9	98.8–99.0
				
*Baseline HC2 positivity threshold, RLU/CO*				
1	95.5	95.3–95.8	95.9	95.7–96.2
2	96.2	95.9–96.4	96.5	96.3–96.8
5	96.7	96.5–96.9	97.1	96.8–97.3
7	96.9	96.7–97.1	97.2	97.0–97.4
10	97.2	96.9–97.4	97.5	97.3–97.7
30	98.0	97.8–98.1	98.3	98.1–98.4
50	98.2	98.0–98.4	98.5	98.3–98.6
100	98.7	98.5–98.8	98.9	98.7–99.0

Abbreviations: BMD=borderline or mild dyskaryosis; CI=confidence interval; CIN=cervical intraepithelial neoplasia (grade 2 or 3 or higher); HC2=hybrid capture 2; hrHPV=high-risk human papillomavirus; RLU/CO=relative light unit/cutoff.

**Table 3:** Relative colposcopy referral rates, relative sensitivity, relative false positivity rate of HC2 RLU/CO thresholds at baseline alone *vs* baseline cytology testing, adjusted for non-attendance at repeat testing

		**End point CIN3+**		**End point CIN2+**	
**Test**	**Relative colposcopy referral rate**	**Relative sensitivity**	**Relative false positivity rate**	**Relative sensitivity**	**Relative false positivity rate**
Baseline cytology (threshold BMD)	Reference	Reference	Reference	Reference	Reference
					
*Baseline HC2 positivity threshold, RLU/CO*					
1	2.82	1.36	3.35	1.50	3.67
2	2.48	1.34	2.89	1.48	3.12
5	2.18	1.31	2.49	1.42	2.65
7	2.06	1.28	2.34	1.40	2.48
10	1.90	1.22	2.14	1.34	2.25
30	1.42	1.08	1.53	1.20	1.55
50	1.24	0.95	1.34	1.05	1.35
100	0.94	0.80	0.99	0.82	1.01

Abbreviations: BMD=borderline or mild dyskaryosis; CIN=cervical intraepithelial neoplasia (grade 2 or 3 or higher); HC2=hybrid capture 2; RLU/CO=relative light unit/cutoff.

**Table 4:** Relative colposcopy referral rates, relative sensitivity, relative false positivity rate of HC2 positivity threshold with baseline triage and repeat testing strategy *vs* baseline cytology testing, adjusted for non-attendance at repeat testing

				**End point CIN3+**		**End point CIN2+**	
**Test**			**Relative colposcopy referral rate**	**Relative sensitivity**	**Relative false positivity rate**	**Relative sensitivity**	**Relative false positivity rate**
Baseline cytology (threshold BMD)			Reference	Reference	Reference	Reference	Reference
							
*Baseline HC2 positivity threshold, RLU/CO*	*Baseline triage test*	*Repeat test at 12 months*					
1	Cytology	Cytology	0.94	1.32	0.80	1.36	0.67
2	Cytology	Cytology	0.89	1.29	0.74	1.34	0.60
5	Cytology	Cytology	0.84	1.25	0.69	1.31	0.60
7	Cytology	Cytology	0.82	1.22	0.68	1.29	0.52
10	Cytology	Cytology	0.79	1.17	0.65	1.23	0.50
30	Cytology	Cytology	0.65	1.02	0.52	1.09	0.38
50	Cytology	Cytology	0.57	0.90	0.45	0.96	0.33
100	Cytology	Cytology	0.46	0.75	0.36	0.74	0.28
							
1	Cytology	Cytology and HC2^a^	1.88	1.36	2.07	1.50	2.12
2	Cytology	Cytology and HC2^a^	1.68	1.34	1.81	1.47	1.82
5	Cytology	Cytology and HC2^a^	1.45	1.30	1.51	1.40	1.48
7	Cytology	Cytology and HC2^a^	1.37	1.27	1.41	1.37	1.37
10	Cytology	Cytology and HC2^a^	1.30	1.21	1.33	1.29	1.30
30	Cytology	Cytology and HC2^a^	0.96	1.05	0.92	1.14	0.84
50	Cytology	Cytology and HC2^a^	0.81	0.93	0.76	1.01	0.68
100	Cytology	Cytology and HC2^a^	0.59	0.76	0.52	0.78	0.46

Abbreviations: BMD=borderline or mild dyskaryosis; CIN=cervical intraepithelial neoplasia (grade 2 or 3 or higher); HC2=hybrid capture 2; RLU/CO=relative light unit/cutoff.

a HC2 RLU/CO threshold as at baseline.
